# Mental Hospital Matters

**Published:** 1911-11-25

**Authors:** 


### MENTAL HOSPITAL MATTERS.

(Administrative and Practical Contributions and Photographs invited.)

### Institution Treatment of the Insane.

THE most important factors in the treatment of the insane in institutions are the regularity of life and the discipline which must of necessity obtain in them. This regularity and discipline are always difficult and in many cases impossible to procure at home.

### HOME TREATMENT.

The patient treated at home always has the knowledge and feeling that he is in his normal surroundings, and has, therefore, the right to freedom of action. In these circumstances he will resent any curtailment of his liberty or restraint on his actions which may become necessary, and may thus retard his recovery by taking a dislike to those who are looking after him and by actively resisting anything that they may have to do for him. In strange surroundings this feeling, that he has a right to do as he likes, is not so strong, especially when he finds himself amongst other patients who are strangers and who, being used to the regulations of the institution, all do the same thing at the same time.

There is then a natural tendency for him to fall into line with the others without demur or resistance, and to do what appears to be the normal thing. This is often very noticeable in patients who are admitted to an institution with a history of refusal of food. These patients, when they see the others go to the table at meal-times in the ordinary way will often follow the example set on being asked to do so, and though they may once or twice require pressing to eat, will afterwards give no further trouble and will take their food well.

### ADVANTAGES OF INSTITUTIONS.

Patients, too, who have been restless and resistive at home generally settle down to quieter ways on admission to an institution, this effect being partly due to the larger space in which they can wander about. Nothing is more irritating to the insane (or to the sane) than to be constantly prevented from going here or there or from doing this, that, or the other. In an institution the rooms and grounds, besides being larger than those of private houses, are specially safeguarded for the care of the insane, so that, whether inside or out of doors, the patient can be allowed to roam about freely and without being restrained in any way to a much greater extent than is possible in more confined places. This fact also gives the reason why sedative drugs are used less often in treating acutely insane patients in institutions than in treating them at home.

### THE PROBLEM OF AMUSEMENT.

The two important items in treatment—amusement and employment—can be supplied more easily and in greater variety in an institution than at home. Entertainments of various kinds, given by people from outside the institution, are the rule at regular intervals during the winter months, and generally alternate with dances; whilst varied amusements and games can also be got up both indoors and outside, and more interest stimulated in them where there are many to take part. Employment can as a rule be given in good variety in institutions, though it does not seem to be supplied and organised as well as it might be. There is always the primary difficulty to be overcome of the patient's unwillingness and possible refusal to follow any organised employment, and this is harder to deal with in private than in pauper patients, since to the latter various little things (*e.g.* tobacco, tea, etc.) may be given as rewards, whilst to the former these are usually supplied by the friends. Private patients, however, will usually help in the household work in the wards of an institution more readily than they will do the same work at home, and their interest can thus be stimulated in something new to them—the life and work in the institution.

### AN ALLEGED DRAWBACK.

It is often put forward as a drawback to institution treatment of the insane that the patient is put in a ward with others worse than himself, and that this is bound to do harm and retard recovery. Experience teaches that this is very rarely the case where proper classification is carried out; indeed, it can be taken almost as an axiom in institution work that when patients point to others and ask how they are to be expected to get better with " people like that " about them, it is certain that they are worse than those pointed to; for it is a curious fact that when this remark is made in nine cases out of ten those pointed to are either recovered or convalescent cases—the most normal patients in the ward.

### A CURIOUS FACT.

When patients get better they cease to make remarks like this. The one wish of the patients who have recovered seems to be to do all they can to help those who are still ill, and after discharge from the institution they will very frequently come back to see those who remain to try to help them. Another curious fact noticed in institutions is that when patients who have been rowdy get better and it is suggested to them that they should go to a quieter ward, they nearly always say that they would sooner remain where they are, as life seems so dull in the quieter wards as to make them bored.

